# Psychiatric Approach in Phantom Erection Postpenectomy Patient

**DOI:** 10.1155/2023/4113455

**Published:** 2023-03-31

**Authors:** Popy Arizona, Erikavitri Yulianti, Izzatul Fithriyah

**Affiliations:** Department of Psychiatry, Dr. Soetomo General Academic Hospital, Universitas Airlangga, Surabaya, Indonesia

## Abstract

**Introduction:**

Phantom limb pain is a pain sensation experienced in the area of the missing body part. The pain generally appears in the first few days after surgery. PLP could occur in teeth, tongue, breast, eyes, rectum, bladder, testicles, and penis. Phantom pain in the penis is not only felt as pain but sometimes as an erection or urination, even after the removal of the penis. *Clinical Case*. A 35-year-old man was referred to the psychiatrist due to phantom erection after undergoing reimplantation of the penis by the urologist. A few days before the referral, he was admitted to the emergency department after a penile amputation that his wife performed. During the recovery phase after the penile reimplantation procedure, the patient worried about his penis' outcome and became depressed. The patient was in severe anxiety and moderate-to-severe depression status. *Treatment*. The patient was given nonpsychopharmacology such as supportive psychotherapy, family psychoeducation, relaxation and marital therapy, and psychopharmacology, such as amitriptyline 12.5 Mg PO two times a day and clobazam 10 Mg PO each day for 3 months. One and a half months later, his anxiety and depression were better.

**Conclusion:**

A psychiatric approach was needed in an amputated limb patient with psychopathologic symptoms. Nonpsychopharmacotherapy and psychopharmacotherapy were needed if the patient had symptoms. Further studies with a large number will be necessary to validate the psychiatric approach in amputated limb patients with psychopathologic symptoms cases.

## 1. Introduction

Phantom limb pain (PLP) was first described by Ambroise Paré in 1552. Phantom limb pain is a pain sensation experienced in the area of the missing body part. The pain generally appears in the first few days after surgery. Many patients, after recovering from an anesthetic agent, feel that the amputated part is still present in its usual place [1–5]. PLP did not occur in impacted limbs only but also in other body parts, such as teeth, tongue, breast, eyes, rectum or bladder, testicles, and penis [6]. Phantom pain in the penis is not only felt as pain but sometimes as an erection or urination, even after the removal of the penis.

The World Health Organization's International Classification of Diseases (ICD) recognizes PLP as a type of chronic pain [7, 8]. Chronic pain after amputation was classified as chronic postsurgical under the code “8E43.00-Phantom limb syndrome” in the chapter “other disorders of the nervous system” in the most recent version of the ICD, ICD-11 [7]. PLP is not classified as a separate diagnosis in the DSM-5-TR (Diagnostic and Statistical Manual of Mental Disorders). However, PLP may be a symptom of another disorder, such as postamputation pain disorder or complex regional pain syndrome (CRPS) [9]. Overall, both the ICD-11 and the DSM-5-TR recognize PLP as a type of pain that can occur in people with specific medical conditions, and they guide its classification and diagnosis.

Phantom erection is one of the psychological concerns in patients treated with penile amputation. After the amputation, the occurrence of phantom sensation is commonly seen. This effect has been observed since the mid-1700s, and most cases do not receive any clinical or therapeutic attention [10]. After amputation, frustration may occur in the patient alongside a loss of confidentiality and emotional strain. The prevalence of psychiatric pathologies, such as depression, anxiety, and mood disorders, is also higher in amputees than in the general population [11]. Thus, this case report describes the psychiatry approach to phantom erection in a postpenectomy patient.

## 2. Case Report

A 35-year-old man was referred to the psychiatry department at Dr. Soetomo General Hospital, Surabaya, due to phantom erection after undergoing reimplantation of the penis by the urology department. A few days before the referral, he was admitted to the emergency department because of a penile amputation that his wife performed. His wife cut his penis because she felt jealous and knew he had an affair with other women. Neither the patient's wife nor the patient was given a history of mental disorders during or before the examination. So the pure patient's wife committed that act out of jealousy of her husband, who had an affair with another woman. The patient and his wife had no legal issues, and the patient did not report his wife's actions to authorities. As a result, it is amicable. The patient has a sibling, but there were no psychiatric disorders in the patient's family, siblings, or parents.

Then, the urology department decided to perform penile reimplantation. The patient was informed that if the reimplantation was not viable and if there was an infection, a penectomy may be performed on the patient. Two days after the penile reimplantation procedure, the patient said he was satisfied with the procedure because he could feel his penis was functioning again, proved by the fact that he had an erection that day. Four days after admission, he was referred to the psychiatry department by the urology department to assess his anxiety and help him to adjust his condition.

At the first meeting with a psychiatrist, the patient complained about pain in his penis. There is no anxiety or depression. There is no hallucination. The patient did not see or hear things that another person did not see or hear. The patient communicated appropriately, orientation was good, the mood was dysphorically affected by pain, the perception was good, motivation was good, psychomotor was good, and no psychopathology symptoms.

Three days after the first meeting, he felt sad and worried that reimplantation was failed. His psychiatric status was depressed, and his mood was affected by anxiety. Then, the psychiatrist performed Beck Depression Inventory (BDI) and Hamilton Anxiety Rating Scale (HARS) psychometry, the result was 29 (severe depression) and 27 (moderate to severe anxiety). He was diagnosed with adjustment disorder with mixed and depressed mood (F43.23) by the psychiatrist. He was suggested to have supportive psychotherapy, family psychoeducation, relaxation therapy, and marital therapy. Marital therapy is a type of psychotherapy aimed at assisting couples in improving their relationship. In the context of penile amputation, marital therapy can assist couples in dealing with the complex emotions and challenges that this traumatic event brings. This therapy focuses on partner communication skills, problem-solving strategies, sexual issues, and conflict resolution. Psychoeducation entails educating and informing individuals and couples about mental health conditions, treatment options, and coping strategies. Psychoeducation can help couples understand the physical and emotional implications of penile amputation and develop coping strategies. In addition, psychopharmacology was also given, such as fluoxetine 10 Mg PO each morning.

**Figure 1:**
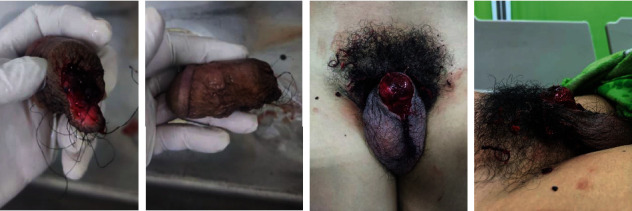
Amputated penile.

A few days after, the penile became necrosis, and it was concluded that the reimplantation failed (Figure 1). The patient was informed that a penectomy must be performed and agreed upon. After the penectomy, the patient thought the suture was off because he could feel a pain sensation on his penis. At the next follow-up by the psychiatrist, he was still depressed. He wanted to go home and recover as soon as possible so he could work again. From a psychiatry point of view, his diagnosis was

- AXIS I: adjustment disorder with anxiety and depressed mood (F43.2)
- AXIS II: avoidance
- AXIS III: phantom limb pain
- AXIS IV: socioeconomic problems
- AXIS V: GAF scale 50

The psychiatrist suggested that the patient continue supportive psychotherapy, family psychoeducation, relaxation, and marital therapy. Marital therapy was performed to help him and his wife rebuild their marriage and compromise with each other. He was given psychopharmacology, such as amitriptyline 12.5 Mg PO two times a day as an antidepressant and analgesic agent and clobazam 10 Mg PO as an antianxiety agent for 3 months.

One and a half months after, the patient and his wife came to the psychiatry department for a follow-up. At the follow-up, the patient said he felt better and started to accept his condition. His BDI and HARS scores were 21 (moderate depression) and 18 (mild to moderate anxiety).

## 3. Discussion

Phantom limb pain (PLP) is a pain sensation experienced in the area of the missing body part [1, 2]. PLP not only includes limbs but also other missing body parts, such as teeth, tongue, breast, eyes, rectum, bladder, testicles, and penis [6]. Before PLP was concluded in the patient, it is important to differentiate it from stump pain, a pain that was felt in the stump of the residual limb [11]. Phantom pain generally appears in the first few days after surgery. Many patients, upon recovering from anesthesia, feel that the amputated part is still present in its usual place [3–5].

The exact mechanism of this phenomenon is still enigmatic. However, some studies said the effects occur due to “cross-activation” between the deafferented cortex and the surrounding areas of the amputated limb. The pathophysiology process of PLP involves complex interactions between the peripheral nervous system and the central nervous system. Amputation of the limb, in this case, the penis, involves an axotomy of the peripheral nervous system that leads to a complete disconnection of peripheral motor and sensory nerve structures. The disconnection may lead to hyperexcitation of sensory neurons, which is produced by inflammatory mediators released by macrophages, Schwann cells, and mast cells, as well as to changes in sodium channels and gene expression [2]. Meanwhile, in the central nervous system, pain is related to unappropriated reorganization in the primary sensory-motor cortex, including changes in motor cortex excitability and peripheral factors, such as nociceptive inputs from the residual limb pain [12, 13].

Phantom erections are associated with pleasurable sensations in contrast to limb loss, which has been associated with painful phantoms for centuries [14]. A case report by Namba et al. suggested that the mechanism of phantom erection involves the persistence of the bulbocavernosus muscle and bulbar corpus spongiosum in the stump of the penis after amputation [15]. Presumably, under normal circumstances, erotic stimuli involve the parietal sensory cortex, and then the penile vasomotor response is elicited, possibly via a descending hypothalamic pathway. It might be deemed as an involuntary, reflexive, or automatic reaction to an erotic thought or sensory perception. When the sensory receptors of the peripheral system are eliminated or amputated, the corresponding cortical circuitry lies dormant and waiting for activation by another type of incoming stimulus. For example, the sexual feeling could be attributed to the feedback process through the parietal cortex, thus causing a phantom erection [16].

A higher prevalence of adverse effects of PLP was reported in several studies on patients with low quality of life, psychological distress, lack of treatment responses, activity limitation, anxiety, and depression [3–5]. The prevalence of psychiatric pathologies, such as depression, anxiety, and mood disorders is also higher in amputees than in the general population [11]. In our case, the patient has moderate anxiety and depression, which means that is more severe than mild, but not severe enough to be classified as severe or disabling. The classification of symptoms is not always clear, and some of the categories may overlap. Furthermore, the severity of symptoms can change over time, and what one person considers moderate anxiety or depression may be different for another. So symptom categorization is important because it helps doctors diagnose problems and develop the best treatment plan for each individual [17]. We also use the DSM-IV-TR multiaxial system to provide a comprehensive and holistic understanding of the patient's mental health and functioning.

Authors encourage performing psychiatric approaches in a patient that has psychopathology symptoms. Reality therapists help patients evaluate and take action by changing what they do and think [18]. Due to the loss of his penis, the patient cannot fulfill his sexual needs. The main purpose of reality therapy is to help the client fulfill the patient's needs, such as survival, love, belonging, power, freedom, and fun based on the actual and current penile amputation [18]. Reality therapists will teach the patient how to make better and more effective decisions and mindset, encouraging him to take charge of their basic needs in their life and maintain his quality of life. The patient was motivated to identify their need to love and to be loved as well as self-worth or internal motivation, rather than external motivation [18].

As a psychiatrist, we provide a variety of therapies, such as supportive psychotherapy, family psychoeducation, relaxation techniques, marital therapy, and psychopharmacology. Supportive psychotherapy can help patients and their families cope with the emotional and psychological effects of surgery. Family psychoeducation can help families understand the physical and emotional changes that come with penile amputation, as well as reduce stigma and promote a supportive environment. Relaxation techniques can help patients manage their feelings on their own. Marital therapy can help patients and their spouses deal with complex emotions and challenges, as well as any related issues that may have contributed to the incident [19–21]. It is generally advised to combine supportive psychotherapy with antidepressant supplements or pharmacotherapy [21]. These therapies can assist patients and their families in managing emotions, dealing with the physical and emotional consequences of surgery, and working toward recovery.

## 4. Conclusion

We conclude from our case report that a psychiatric approach was needed in amputated limb patients with psychopathologic symptoms. Psychiatric treatment might include nonpsychopharmacotherapy and psychopharmacotherapy. Further studies with a large number will be necessary to validate the psychiatric approach in amputated limb patients with psychopathologic symptoms cases.

## Data Availability

The data used to support the findings of this study are available from the corresponding author upon request.
